# Decreased left atrial function in obesity patients without known cardiovascular disease

**DOI:** 10.1007/s10554-022-02744-3

**Published:** 2022-10-28

**Authors:** Y. S. Aga, D. Kroon, S. M. Snelder, L. U. Biter, L. E. de Groot-de Laat, F. Zijlstra, J. J. Brugts, Bas M. van Dalen

**Affiliations:** 1 Department of Cardiology, Franciscus Gasthuis & Vlietland, Kleiweg 500, 3045 PM Rotterdam, The Netherlands; 2 Department of Bariatric Surgery, Franciscus Gasthuis & Vlietland, Rotterdam, The Netherlands; 3 Department of Cardiology, Thoraxcenter, Erasmus University Medical Center, Rotterdam, The Netherlands; 4 Department of Cardiology, Maasstad Hospital, Rotterdam, The Netherlands

**Keywords:** Obesity, Left atrial strain, Echocardiography, Heart failure with preserved ejection fraction

## Abstract

Obesity is a risk factor for heart failure with preserved ejection fraction (HFpEF). We hypothesized that assessment of left atrial (LA) strain may be useful to reveal precursors of HFpEF in obesity patients. Echocardiograms of obesity patients without known cardiovascular disease who underwent bariatric surgery, and echocardiograms of age- and gender matched controls were analyzed. The echocardiogram was repeated 1 year after bariatric surgery. LA reservoir strain (LASr), LA conduit strain (LAScd), and LA contractile strain (LASct) were measured. 77 Obesity patients were compared with 46 non-obese controls. Obesity patients showed a significantly decreased LA function compared with non-obese individuals (LASr 32.2% ± 8.8% vs. 39.6% ± 10.8%, p < 0.001; LAScd 20.1% ± 7.5% vs. 24.9% ± 8.3%, p = 0.001; LASct 12.1% ± 3.6% vs. 14.5% ± 5.5%, p = 0.005). There was no difference in prevalence of diastolic dysfunction between the obesity group and controls (9.1% vs. 2.2%, p = 0.139). One year after bariatric surgery, LASr improved (32.1% ± 8.9% vs. 34.2% ± 8.7%, p = 0.048). In the multivariable linear regression analysis, BMI was associated with LASr, LAScd, and LASct (β =  − 0.34, CI − 0.54 to − 0.13; β =  − 0.22, CI − 0.38 to − 0.06; β =  − 0.10, CI − 0.20 to − 0.004). Obesity patients without known cardiovascular disease have impairment in all phases of LA function. LA dysfunction in obesity may be an early sign of cardiac disease and may be a predictor for developing HFpEF. LASr improved 1 year after bariatric surgery, indicating potential reversibility of LA function in obesity.

## Introduction

Obesity affects around 650 million adults worldwide and the prevalence is increasing [1]. Obesity is a major risk factor for heart failure with preserved ejection fraction (HFpEF) [2, 3]. A one-unit increase in body mass index (BMI) is associated with a 34% increased risk of future HFpEF, and more than 80% of HFpEF patients are either overweight or obese [4, 5]. In the recent years, left atrial (LA) dysfunction has been increasingly recognized as an important parameter in HFpEF [6, 7]. Obesity causes LA dysfunction due to systemic inflammation, expansion of epicardial adipose tissue, and chronic volume overload, all factors that can favor the development of diastolic dysfunction and HFpEF [8–10].

Traditionally, the LA is evaluated by using LA volume indexed (LAVI) to body surface area (BSA), which is widely used and recommended in guidelines as a criterion for diastolic function and HFpEF [11]. However, LAVI in obesity is unsuitable as indexing to BSA overcorrects LA volume and thus does not reflect a proper evaluation of the LA [12]. Recent studies have shown that LA strain provides superior information over the use of LAVI and left ventricular global longitudinal strain (LV GLS), and has better correlation with invasive filling pressures than LAVI [13–17]. Furthermore, LA strain independently predicts incident HFpEF and appears to be altered before traditional parameters of HFpEF can be detected [18–21]. Considering the limited value of LAVI in obesity, we hypothesized that LA strain may be especially useful in these patients. Additionally, symptoms such as dyspnea and edema, findings at physical examination, and brain natriuretic peptides (BNP) might be less specific and/or sensitive for heart failure in patients with obesity [10, 22], another argument stressing the need for improvement of objective parameters of HFpEF in obesity.

The aim of our study was to (1) determine whether LA function measured by LA strain in in obesity patients without known cardiovascular disease differs from non-obese individuals and (2) to determine whether LA function improves 1 year after bariatric surgery in obesity patients.

## Methods

For this study, the CARDIOBESE (The CARdiac Dysfunction In Obesity: Early Signs Evaluation) database was used. The protocol of the CARDIOBESE study has been described before [23]. Briefly, the CARDIOBESE study recruited a cohort of 100 patients with obesity aged 35 to 65 years, with a BMI ≥ 35 kg/m^2^ who were referred for bariatric surgery at The Franciscus Gasthuis and Vlietland and Maasstad Hospital, both in Rotterdam, the Netherlands. Patients with a suspicion of or known cardiovascular disease were excluded. Fifty age- and gender-matched non-obese (BMI ≤ 30 kg/m^2^) controls without a suspicion of or known cardiovascular disease were enrolled. The study protocol was approved by the ethics committee and participants provided written informed consent.

### Echocardiography and strain analyses

**Fig. 1:**
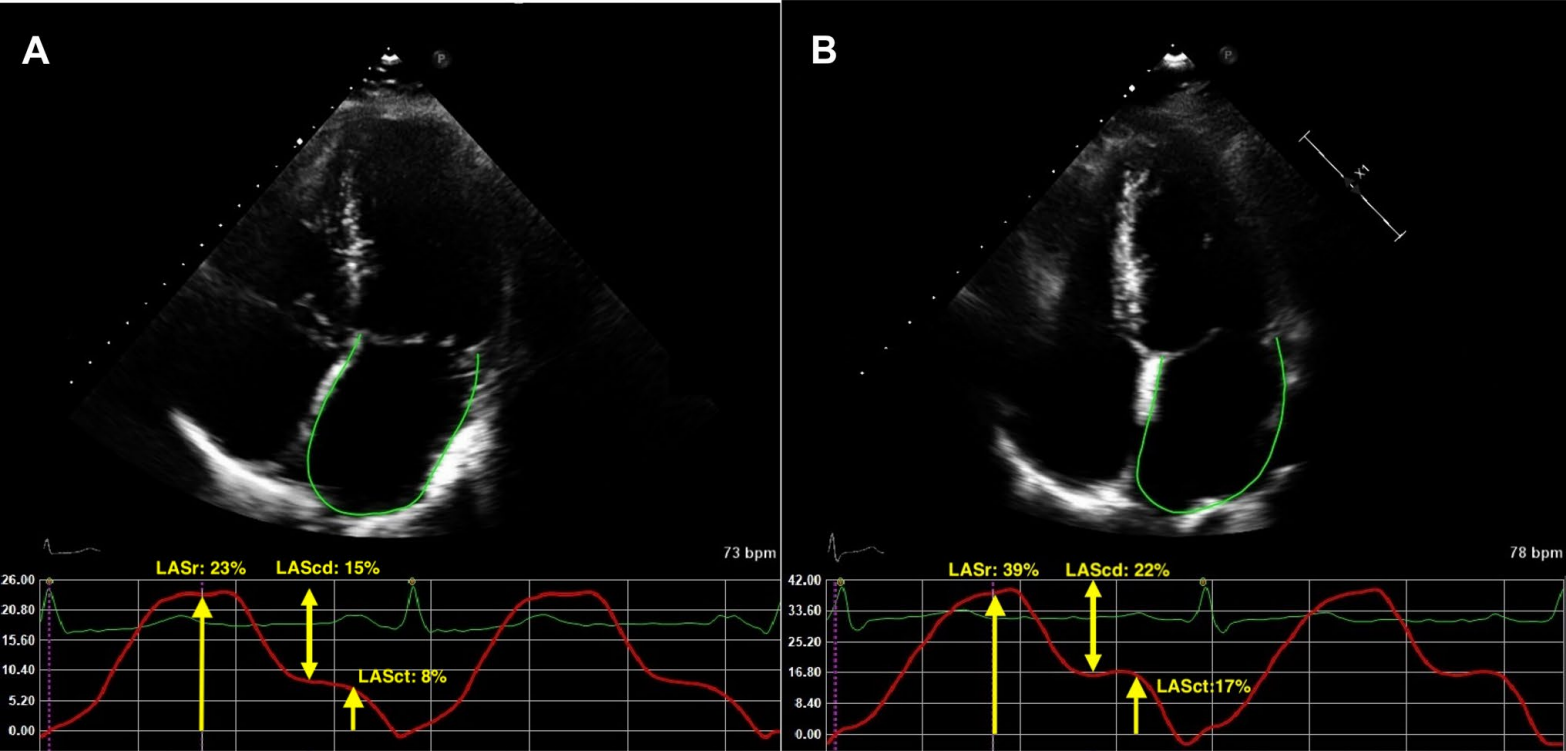
**A** Example of LA strain curve in a patient with obesity. **B** Example of LA strain curve in a control subject obesity. *LASr* left atrial reservoir strain, *LAScd* left atrial conduit strain, *LASct* left atrial contractile strain

Conventional and speckle tracking echocardiography was performed on all participants at baseline. In the obesity group echocardiography was repeated 1 year after bariatric surgery. Two-dimensional greyscale harmonic images were obtained in the left lateral decubitus position using a commercially available ultrasound system (EPIQ 7, Philips, The Netherlands), equipped with a broadband (1–5 MHz) X5-1 transducer. All acquisitions and measurements were performed according to the current guidelines [24, 25]. LA strain was measured with speckle tracking and analyzed offline with dedicated software (TomTec-Arena, integrated in Sectra IDS7). LA strain measurements were performed by a single observer (D.K.) who was blinded to clinical data. The apical 4-chamber view was used preferably for the analysis. LA endocardial borders were automatically traced using end-diastole as reference. When tracking was suboptimal, fine-tuning was performed manually. If the 4-chamber view was of poor image quality, the 2-chamber view was used. Patients with images of insufficient quality to perform LA strain analysis were excluded. LA function was described according to the three phases of the LA cycle: LA reservoir strain (LASr) which starts at the end of ventricular diastole (mitral valve closure) and continues until mitral valve opening, LA conduit strain (LAScd) which occurs from the time of mitral valve opening through diastasis until the onset of LA contraction, and LA contractile strain (LASct) which occurs from the onset of LA contraction until the end of ventricular diastole (mitral valve closure). LASr, LAScd, and LASct were computed in all participants. All strain values are reported as absolute values for improved readability and data interpretation [26]. An example of LAS measurement in a patient without obesity and with obesity is shown in Fig. 1.

### Statistical analysis

Normally distributed data are presented as means and standard deviation, skewed data as medians and inter‐quartile range, and categorical variables as percentages and frequencies. Continuous variables were compared between obesity patients and controls using the independent Student T-test in case of normally distributed data and the Mann–Whitney U test for non-normally distributed data. For the comparison before and 1 year after bariatric surgery, the dependent T-test was used for normally distributed data and the Wilcoxon signed rank for non-normally distributed data. Categorical data was analyzed with the Chi-square test and the McNemar’s test for respectively normally and non-normally distributed data. To determine whether BMI was associated with LASr, LAScd, and LASct, independent of potential confounders, univariable and multivariable linear regression analysis were performed. Variables were included in the model at a statistical level p-value of < 0.05 and performed using the enter method in linear regression. In the multivariable model we included age, gender, diabetes mellitus, hypertension, obstructive sleep apnea syndrome (OSAS), because of the potential clinical relation with the outcome variable. For the linear regression we report coefficients, 95% confidence intervals (CI) and p-values. Analyses were performed using SPSS Statistical Package version 28.0.

## Results

A total of 100 patients with obesity and 50 controls were included in the CARDIOBESE study. Out of these, 77 patients with obesity and 46 controls had sufficient image quality to quantify LA strain and were thus included in the analysis. Of the patients with obesity, 72 underwent bariatric surgery, of whom 59 patients were included in the analysis. The remaining patients were excluded because of insufficient image quality to measure LA strain. Clinical characteristics of the study population are shown in Table 1.

**Table 1:** Clinical characteristics of the study population

	Comparison between controls and obesity patients			Comparison between patients with obesity before and 1 year after bariatric surgery		
	Controls (N = 46)	Obesity patients (N = 77)	p-value	Baseline (N = 59)	One-year after bariatric surgery (N = 59)	p-value
Age (years)	49.5 ± 9.4	48.1 ± 7.1	0.379	48.1 ± 7.2	–	–
Female [n (%)]	33 (71.7)	59 (76.6)	0.550	46 (78.0)	–	–
BMI (kg/m^2^)	25.1 (22.0–27.5)	42.5 (41.6–43.4)	< 0.001	41.2 (39.5–46.1)	28.4 (24.7–31.2)	< 0.001
Systolic BP (mmHg)	126.7 ± 10.4	141.2 ± 20.5	< 0.001	140.0 (129.0–157.0)	129.5 (116.5–143.3)	0.004
Diastolic BP (mmHg)	76.0 ± 8.6	79.6 ± 10.9	0.067	79.0 (73.0–88.0)	80.0 (74.0–86.0)	0.248
Diabetes mellitus [n (%)]	0	17 (22.1)	< 0.001	13 (22.0)	5 (8.5)	0.008
Hypertension [n (%)]	3 (6.5)	23 (29.9)	< 0.001	18 (30.5)	9 (15.3)	0.064
Hypercholesterolaemia [n (%)]	5 (10.9)	16 (20.8)	0.134	12 (20.3)	6 (10.2)	0.146
OSAS [n (%)]	1 (2.2)	9 (11.7)	0.028	6 (10.2)	2 (3.4)	0.031
Beta-blockers [n (%)]	0	6 (7.8)	0.013	4 (6.8)	3 (5.1)	1.000
RAS-inhibitor [n (%)]	2 (4.3)	18 (23.4)	0.001	13 (22.0)	5 (8.5)	0.021
Statins [n (%)]	3 (6.5)	16 (20.8)	0.018	14 (23.7)	7 (11.9)	0.039
Diuretics [n (%)]	1 (2.2)	14 (18.2)	0.002	11 (18.6)	5 (8.5)	0.070

Normally distributed data are presented as mean ± s.d., non-normally distributed data are presented as median (25th interquartile–75th interquartile), categorical data are presented as n (%)

*BMI* body mass index, *BP* blood pressure, *OSAS* obstructive sleep apnea syndrome, *RAS* renin–angiotensin-system

### Comparison between non-obese controls and obese patients

As presented in Table 1, patients with obesity showed several differences compared to non-obese controls. Obesity patients had a higher systolic blood pressure (141.2 mmHg ± 20.5 mmHg vs. 126.7 mmHg ± 10.4 mmHg, p < 0.001), and comorbidities, such as diabetes mellitus, hypertension, and OSAS were more frequently present in the obesity group (22.1% vs. 0%, p < 0.001; 29.9% vs. 6.5%, p < 0.001; 11.7% vs. 2.2%, p = 0.028 respectively). Obesity patients more often used beta-blockers, RAS-inhibitors, statins and diuretics.

Differences in echocardiographic parameters are shown in Table 2. There was no difference in LAVI between the two groups (26.1 ml/m^2^ ± 6.1 ml/m^2^ vs. 25.8 ml/m^2^ ± 6.7 ml/m^2^, p = 0.809). As for parameters of LV diastolic function, obese patients had lower E/A ratio (1.0 ± 0.25 vs. 1.2 ± 0.3, p < 0.001) and lower lateral e′ velocity (11.0 cm/s ± 3.2 cm/s vs. 13.4 cm/s ± 6.5 cm/s, p = 0.007). There was no significant difference in the prevalence of diastolic dysfunction (2.2% vs. 9.1%, p = 0.087).

**Table 2:** Echocardiographic parameters of the study population

	Comparison between controls and patients with obesity			Comparison between patients with obesity before and 1 year after bariatric surgery		
	Controls (N = 46)	Patients with obesity (N = 77)	p-value	Baseline (N = 59)	One-year after bariatric surgery (N = 59)	p-value
LVM (g)	146.1 ± 40.9	190.0 ± 69.8	< 0.001	184.3 ± 74.4	156.8 ± 66.3	0.004
LVM index (g/m^2^)	74.3 ± 18.9	77.3 ± 24.5	0.470	75.1 ± 25.8	77.8 ± 24.7	0.41
LVEDD (mm)	44.9 ± 5.1	49.7 ± 6.0	< 0.001	49.3 ± 6.4	48.5 ± 5.4	0.33
E/A ratio	1.2 ± 0.3	1.0 ± 0.3	< 0.001	1.0 (0.9–1.1)	1.1 (0.9–1.2)	0.047
Lateral e′ velocity (cm/s)	13.4 ± 6.5	11.0 ± 3.2	0.007	11.0 ± 3.4	12.1 ± 3.2	0.003
E/e′ ratio	8.6 ± 2.1	9.1 ± 2.3	0.278	9.1 ± 2.4	8.7 ± 2.3	0.27
Deceleration time (s)	0.2 ± 0.03	0.18 ± 0.04	0.578	0.19 ± 0.04	0.19 ± 0.05	0.87
TR velocity (cm/s)	137.8 ± 69.8	123.7 ± 54.5	0.255	105.9 (89.6–140.5)	194.8 (108.3 ± 223.6)	< 0.001
LA volume index (ml/m^2^)	26.1 ± 6.1	25.8 ± 6.7	0.809	25.1 ± 6.5	28.5 ± 7.3	0.001
LV ejection fraction (%)	65.3 ± 5.5	57.1 ± 6.7	< 0.001	55.8 ± 6.5	57.3 ± 7.0	0.21
GLS (%)	− 20.1 ± 1.5	− 16.7 ± 2.8	< 0.001	− 16.6 ± 2.7	− 18.2 ± 3.1	0.003
Diastolic function [n (%)]						
Normal	45 (97.8)	70 (90.9)	0.139	53 (89.8)	55 (93.2)	0.73
Indeterminate	0 (0)	0 (0)		0 (0)	0 (0)	
Dysfunction	1 (2.2)	7 (9.1)		6 (10.2)	4 (6.8)	

Normally distributed data are presented as mean ± s.d., non-normally distributed data are presented as median (25th interquartile–75th interquartile), categorical data are presented as n (%)

*LVM* left ventricular mass, *LVEDD* left ventricular end-diastolic diameter, *TR* tricuspid regurgitation, *LA* left atrial, *LV* left ventricular, *GLS* global longitudinal strain

### Comparison between obese patients at baseline and 1 year after bariatric surgery

Table 1 shows the differences in clinical characteristics for patients with obesity from baseline to 1 year after bariatric surgery. Gastric bypass was the most common type of bariatric surgery in this group (54.2%), followed by gastric sleeve (35.6%) and mini bypass surgery (10.2%). There was a significant reduction in BMI [41.2 kg/m^2^ (39.5–46.1 kg/m^2^) vs. 28.4 kg/m^2^ (24.7–31.2 kg/m^2^), p < 0.001] and improvement of systolic blood pressure 1 year after bariatric surgery [140.0 mmHg (129.0–157.0 mmHg) vs. 129.5 mmHg (116.5–143.3 mmHg), p = 0.004]. Furthermore, the rates of diabetes mellitus and OSAS improved significantly (22.0% vs. 8.5%, p = 0.008; 10.2% vs. 3.4%, p = 0.031). Also, there was less use of RAS-inhibitors and statins after bariatric surgery (22.0% vs. 8.5%, p = 0.021; 23.7% vs. 11.9%, p = 0.039).

As for echocardiographic parameters (Table 2), E/A ratio and lateral e′ velocity both showed a significant change [1.0 (0.9–1.1) vs. 1.1 (0.9–1.2), p = 0.047; 11.0 cm/s ± 3.4 cm/s vs. 12.1 cm/s ± 3.2 cm/s, p = 0.003]. Remarkably, LAVI increased 1 year after bariatric surgery (25.1 ml/m^2^ ± 6.5 ml/m^2^ vs. 28.5 ml/m^2^ ± 7.3 ml/m^2^, p < 0.001). There was no difference in prevalence of diastolic function (10.2% vs. 6.8%, p = 0.727).

### LA function in obesity

**Fig. 2:**
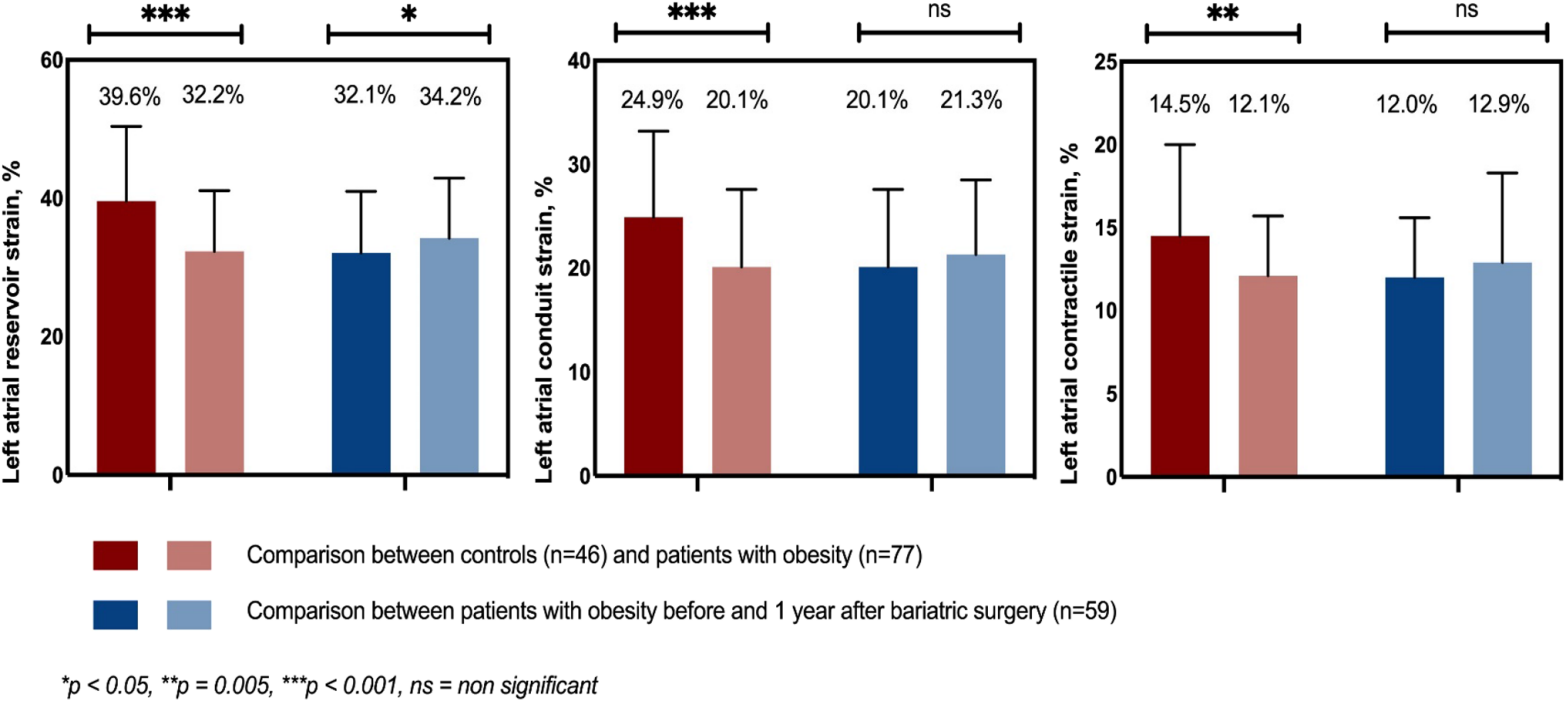
Left atrial stain in patients with obesity before and after bariatric surgery, and in non-obese controls

Differences in LA function measured by LA strain are shown in Fig. 2. Obesity patients had significantly reduced LA strain in all phases of the LA cycle compared with non-obese controls (LASr 32.2% vs. 39.6%, p < 0.001; LAScd 20.1% vs. 24.9%, p < 0.001; LASct 12.1% vs. 14.5%. p = 0.005).

**Fig. 3:**
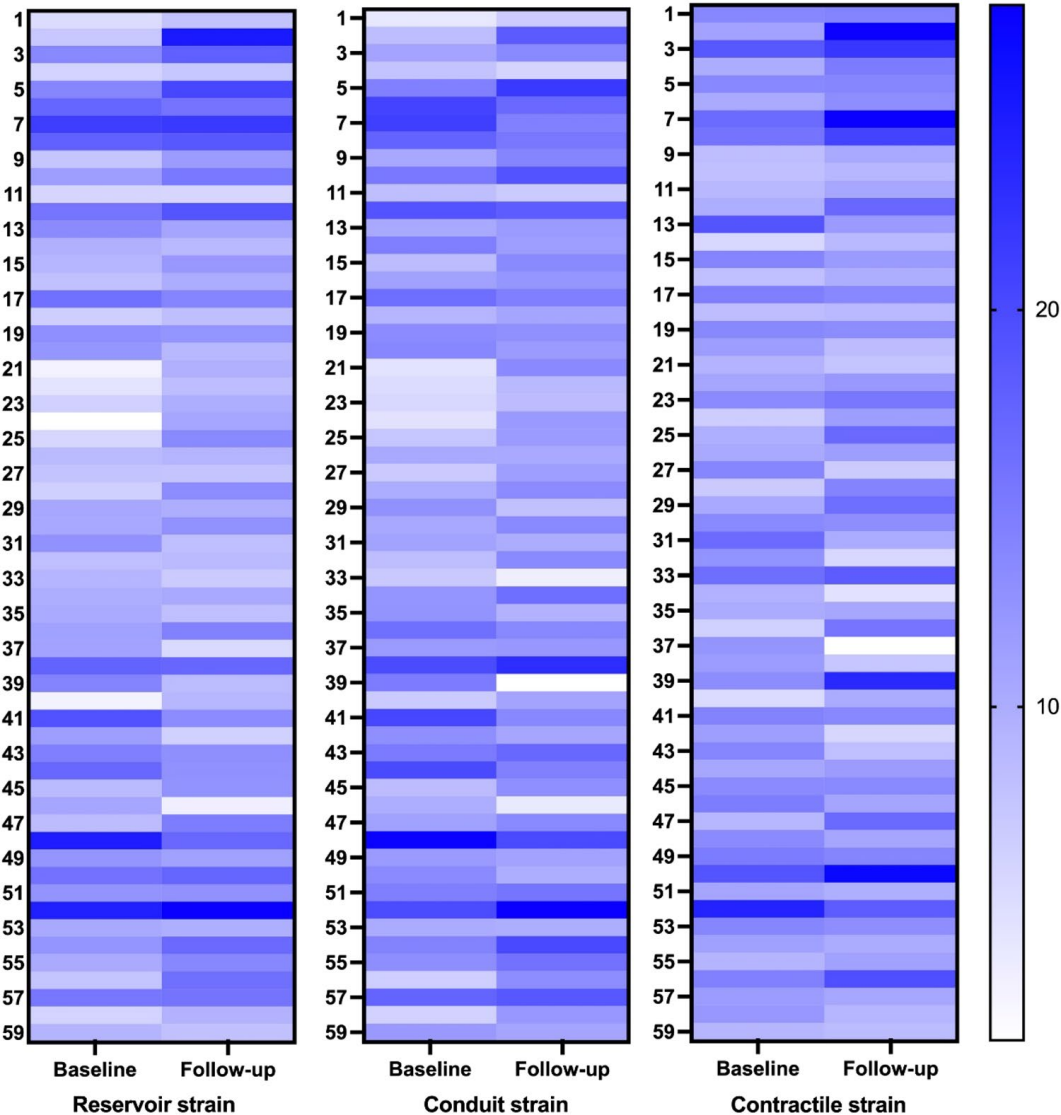
LASr, LAScd, and LASct at individual level for the obesity bariatric surgery group at baseline and 1 year follow-up

In the obese bariatric surgery group, LASr improved significantly 1 year after bariatric surgery (32.1% vs. 34.2%, p = 0.048, Fig. 2). LAScd and LASct showed a tendency towards improvement after bariatric surgery, but did not reach statistical significance. Figure 3 shows the changes in LASr, LAScd, and LASct at individual level for the obese bariatric surgery group.

### Relation between BMI and LA function

Results of the linear regression are presented in Table 3. In simple linear regression, BMI was significantly associated with a LASr, LAScd, and LASct (β − 0.39, 95% CI − 0.57; − 0.20, p < 0.001; β − 0.25, 95% CI − 0.40; − 0.10, p = 0.001; β − 0.12, 95% CI − 0.21; − 0.04, p = 0.005). In a multivariable model, including age, gender, BMI, diabetes mellitus, OSAS, hypertension, beta-blocker use, RAS-inhibitor use, diuretics use, and statin use, an increase in BMI remained significantly associated with a decrease in all three components of LA function (LASr β − 0.34, 95% CI − 0.54; − 0.13, p = 0.002; LAScd β − 0.22, 95% CI − 0.38; − 0.06, p = 0.008; LASct β − 0.10, 95% CI − 0.20; − 0.004, p = 0.041).

**Table 3:** Multivariable linear regression: association of body mass index with left atrial reservoir strain

Dependent variable	Univariable^a^			Multivariable^b^		
	Coefficient	95% CI	p-value	Coefficient	95% CI	p-value
LASr	− 0.39	− 0.57; − 0.20	< 0.001	− 0.34	− 0.54; − 0.13	0.002
LAScd	− 0.25	− 0.40; − 0.10	0.001	− 0.22	− 0.38; − 0.06	0.008
LASct	− 0.12	− 0.21; − 0.04	0.005	− 0.10	− 0.20; − 0.004	0.041

Dependent variable: left atrial reservoir strain

*CI* confidence interval, *BMI* body mass index, *OSAS* obstructive sleep apnea syndrome, *RAS* renin–angiotensin-system

^a^Univariable analysis included BMI

^b^Multivariable analysis included: age, gender, BMI, diabetes mellitus, OSAS, hypertension, beta-blocker, RAS-inhibitor, diuretics, statin

## Discussion

In the present study we have demonstrated that obesity patients without known cardiovascular disease have significantly decreased LA function in all three LA functional components compared to non-obese controls. There was no difference in prevalence of diastolic dysfunction as assessed by the current guidelines, suggesting that LA dysfunction occurs before diastolic dysfunction may be recognized by conventional parameters in obesity patients. An increase in BMI was significantly associated with a decrease in all three components of LA function, confirming that obesity plays an important role in LA dysfunction. LA dysfunction measured with LA strain could be an important parameter of diastolic dysfunction and predictor of HFpEF in obesity. In addition, LASr improved 1 year after bariatric surgery, reflecting positive effects of weight reduction and associated metabolic improvements.

### Identifying diastolic dysfunction and HFpEF in obesity

Obese individuals without known cardiovascular disease often have signs of subclinical cardiac dysfunction [27, 28] and are at greater risk for developing HFpEF [2, 3, 29]. Obesity causes hemodynamic changes, inflammation, and expansion of epicardial adipose tissue that lead to LA myopathy and LA dysfunction which can form a substrate for HFpEF [8, 30, 31]. LA dysfunction measured by strain could be an early marker of subclinical dysfunction and predictor for HFpEF in patients with obesity, but current guidelines do not recommend the use of LA strain in diagnosing HFpEF [11]. In obesity, identifying and recognizing HFpEF is particularly challenging, as signs and symptoms of HFpEF are often attributed to the extra weight and/or other comorbidities that are common in obesity [10]. Moreover, the use of BNP in obesity as diagnostic and prognostic biomarker is hampered, due to the inverse relationship between BMI and BNP [22]. All of this underlines the potential added value of LA strain in obesity.

The use of LAVI as a criterion for diastolic dysfunction and HFpEF in obesity is unsuitable because of the use of BSA as indexation. BSA is disproportionally driven by an increase in fat mass and the use of BSA leads to an overcorrection in obesity [12]. The results of our study are consistent with this notion as is reflected by the observation that LAVI paradoxically increased after bariatric surgery. Furthermore, our results demonstrate that LA impairment is apparent in obesity and that this subclinical cardiac dysfunction would have remained largely unidentified with assessment of diastolic function according to the current guidelines, as is shown by the comparable proportion of obese and non-obese individuals with diastolic dysfunction. In addition, we found a significant improvement of LASr after bariatric surgery, while other diastolic function parameters showed a trend towards improvement, but did not all reach statistical significance. An explanation for this observation could be that the effects of obesity on left atrial function are related to other processes, such as expansion of epicardial adipose tissue and systemic inflammation, and that these adverse effects are not captured by conventional diastolic function parameters. This observation emphasizes that LA strain could have beneficial diagnostic and prognostic value in obesity.

In addition to the observation that patients with obesity have impairment in LA function, we also demonstrated that an increase in BMI was significantly associated with a decrease in all three components of LA function, after adjusting for confounders. However, it should be noted that various factors, such as hypertension, diabetes, and use of cardio-protective medication that ameliorate systemic inflammation, can also potentially influence LA function [32–35]. Thus, it cannot be stated that BMI is the sole explanation for impairment in LA function. Nonetheless, after adjusting for these confounders in our study, LA strain remained significantly associated with BMI, which supports the notion that obesity is related to LA dysfunction.

### LA function in obesity, comparison with other studies

In our analyses, we observed that LASr, LAScd and LASct were significantly reduced in patients with obesity compared to a non-obese control group. Few prior studies have assessed LA function in obesity with speckle tracking echocardiography [36–38]. Findings comparable to our results were reported in a sample consisting of young adolescents with obesity [38]. A study that compared LA function in diabetic patients with and without obesity, found a decreased LASr and LASct in patients with obesity with diabetes [37]. In a larger sample size, Chirinos et al. found lower LASr and LAScd in patients with obesity, but a slightly higher LASct in the obesity group compared to normal weight subjects [36]. A comparison between their study and ours shows that our population had a higher BMI (42.5 kg/m^2^ vs. 32.7 kg/m^2^), which could mirror a more progressive systemic inflammation and atrial myopathy that is reflected in reduced LASct. This is however speculative and further studies are needed for a definite explanation.

### LA function and the effect of bariatric surgery

LASr significantly increased 1 year after bariatric surgery. We did not find significant improvement of LAScd and LASct. Strzelcyk et al. observed similar results and reported a significant increase in LASr and LAScd after bariatric surgery, and a decrease in LASct [39]. The authors explained the decrease in LASct in their study mechanistically as an improvement of early LV diastolic filling that may lead to a relative decrease of the contribution of atrial contraction [39]. Bariatric surgery leads to complex metabolic and hemodynamic changes. Studies have demonstrated that gastric bypass leads to more favorable outcomes when compared to gastric sleeve in terms of improvement of comorbidities, such as diabetes mellitus, and improvement of LV function [40, 41]. In our study, the majority of patients underwent gastric bypass surgery, and we observed that LASr improved 1 year after bariatric surgery. It is uncertain how the type of surgery affects LA function and whether the type of surgery had a role in these improvements in our population. Nonetheless, the observation that LA function can improve after bariatric surgery is promising and might indicate reversibility of LA dysfunction.

### Study limitations

The study has some limitations. First of all, LA strain analysis requires good image quality and not all our subjects had analyzable LA images, which may have affected the identified proportion of LA dysfunction. Secondly, our population consisted of patients who underwent bariatric surgery and contained a large number of women, which could have biased the results. However, around 80% of patients who undergo bariatric surgery are female [42], which explains the high percentage of females in our study. Thirdly, our study included bariatric patients with a BMI ≥ 35 kg/m^2^. It is undetermined whether our results also apply to patients with a BMI ≥ 30 kg/m^2^ < 35 kg/m^2^. Lastly, our population consisted of obesity patients without cardiovascular disease. We did not include patients with HFpEF and our findings therefore only represent patients with obesity and subclinical cardiac dysfunction and not HFpEF patients. We did however identify that LA dysfunction is apparent at a relatively early stage in patients with obesity, and we postulate that LA strain might therefore be useful as an early sign of cardiac disease and a predictor for HFpEF in obesity.

## Conclusion

Obesity patients without known cardiovascular disease have impairment in all phases of LA function. Our findings suggest that LA dysfunction in obesity occurs before diastolic dysfunction, assessed by conventional echocardiographic parameters, may become apparent. Considering the difficult diagnosis of HFpEF in obesity patients due to the relatively limited value of history taking, physical examination, BNP and LAVI measurement, assessment of LA strain could have important added value in identifying these patients at higher risk at an early stage. Finally, our results indicate that LA function can improve after bariatric surgery, indicating potential reversibility of LA function in obesity.
